# Rare Case of Pediatric Post-transplant Lymphoproliferative Disorder Presenting With Pleural Masses Complicated by Pleural Effusions

**DOI:** 10.14309/crj.0000000000001158

**Published:** 2023-09-23

**Authors:** Erini Nessim Kostandy, David Wan, Essam Imseis

**Affiliations:** 1 Department of Pediatrics, Division of Gastroenterology, The University of Texas Health Science Center at Houston, Houston, TX; 2 Department of Diagnostic and Interventional Imaging, McGovern Medical School, University of Texas, Houston, TX; 3 Department of Pediatrics, Division of Gastroenterology, The University of Texas Health Science Center at Houston John P and Katherine G McGovern Medical School, Houston, TX

**Keywords:** post transplant lymphoproliferative disorder, pediatric liver transplant, liver transplant complications, tacrolimus, pleural masses, pediatric transplant, pediatric immune suppression

## Abstract

Post-transplant lymphoproliferative disorder is a complication in organ transplant recipients characterized by uncontrolled proliferation of B-lymphocytes, occurring in 6% of pediatric patients, with risk factors including primary Epstein-Barr virus infection, intensity of immunosuppression, and cytomegalovirus infection. The clinical symptoms are often nonspecific, and it is associated with a high mortality rate if left untreated. We describe a rare case of post-transplant lymphoproliferative disorder who presented with pleural-based masses resulting in pleural effusions.

## INTRODUCTION

Post-transplant lymphoproliferative disorder (PTLD) is a complication in organ transplant recipients characterized by uncontrolled proliferation of B-lymphocytes, occurring in 6% of pediatric patients, with risk factors including primary Epstein-Barr virus (EBV) infection, intensity of immunosuppression, and cytomegalovirus (CMV) infection. The clinical symptoms are often nonspecific, and it is associated with a high mortality rate if left untreated.^1–3^ We describe a rare case of PTLD who presented with pleural-based masses resulting in pleural effusions.

## CASE REPORTS

The patient is a 10-year-old girl with a history of orthotopic liver transplant at 9 months of age due to biliary atresia and a failed Kasai procedure, later complicated by ischemic cholangiopathy resulting in retransplantation at the age of 8 years. She was first found to have positive EBV loads 2 months before retransplantation, suggestive of an acute infection at that time, with a peak viral load of 7,830 copies/mL. The immediate post-transplant EBV peak viral load was 135,739 copies/mL. Before and after retransplant, she was CMV-seronegative. The donor liver was CMV-seropositive and EBV-seronegative. After retransplant, she continued to have intermittent low-level EBV viremia with viral loads ranging from undetectable to <4,000 copies/mL. She received valganciclovir for CMV prophylaxis for 6 months after transplant.

Before retransplant, her immunosuppression regimen consisted of tacrolimus 1 mg twice daily, with serum tacrolimus levels ranging between 4.0 and 6.0 ng/mL. After retransplant, her immunosuppression included induction with 500 mg intravenous methylprednisolone, followed by maintenance with oral prednisone 10 mg daily and tacrolimus 2 mg twice daily, with serum tacrolimus levels ranging from 4.0 to 10.0 ng/mL.

Her retransplant course was initially unremarkable; however, within 1 year, she had intermittent fevers and neutropenia for several months, which had been extensively investigated with no definitive etiology. The febrile episodes self-resolved within 1–2 days, and she was otherwise asymptomatic. Symptoms were attributed to possible transient viral infections and treatment with tacrolimus. The patient also developed microcytic anemia 6 months after transplant, with her hemoglobin gradually dropping to 6.5 g/dL. Given concern for the bicytopenia being partly due to treatment with valganciclovir, the medication was discontinued, with improvement in anemia; however, her neutropenia persisted.

Twenty-seven months after retransplantation, she presented to the emergency department with fever and dry cough. Her initial physical examination was remarkable for mild tachypnea. She was admitted for a sepsis workup and treatment with broad-spectrum antibiotics. Laboratory test results showed leukopenia (2.2 K/CCM) and neutropenia (absolute neutrophil count of 100 cells/μL). Chest X-ray showed small bilateral pleural effusions. Serum EBV and CMV polymerase chain reaction were negative.

**Figure 1.**
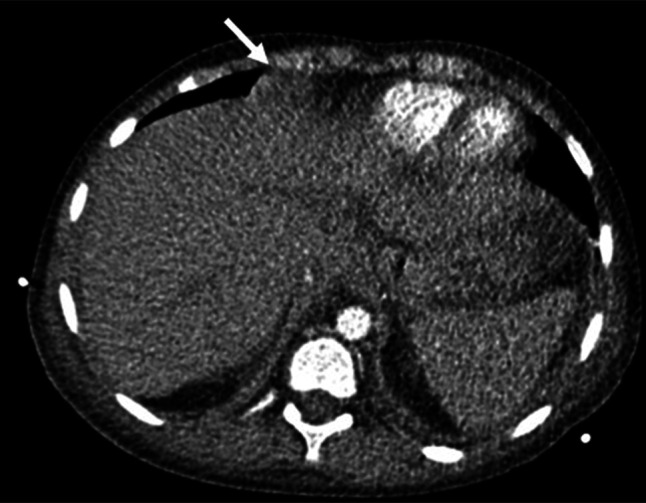
Contrast-enhanced CT scan of a 10-year-old girl after liver transplant showed 1.0 × 1.1 × 2.6 cm right supradiaphragmatic pleural lesion. CT, computed tomography.

**Figure 2.**
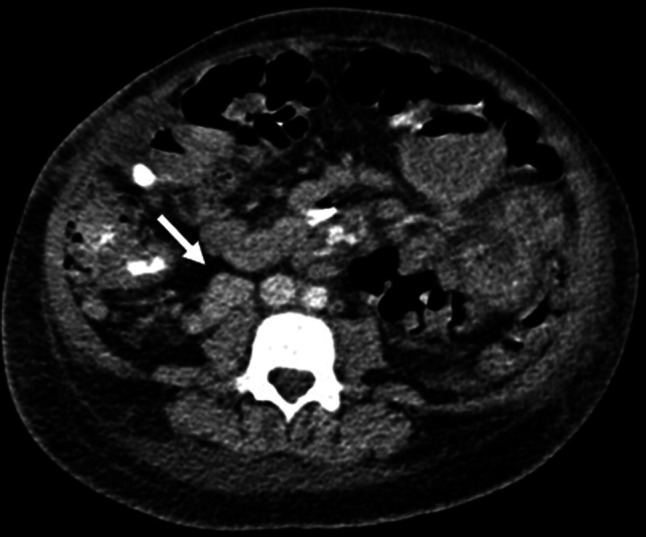
Contrast-enhanced computed tomography scan of a 10-year-old girl after liver transplant showed a 1.7 × 3.3 × 1.5 cm right retroperitoneal lymph node.

**Figure 3.**
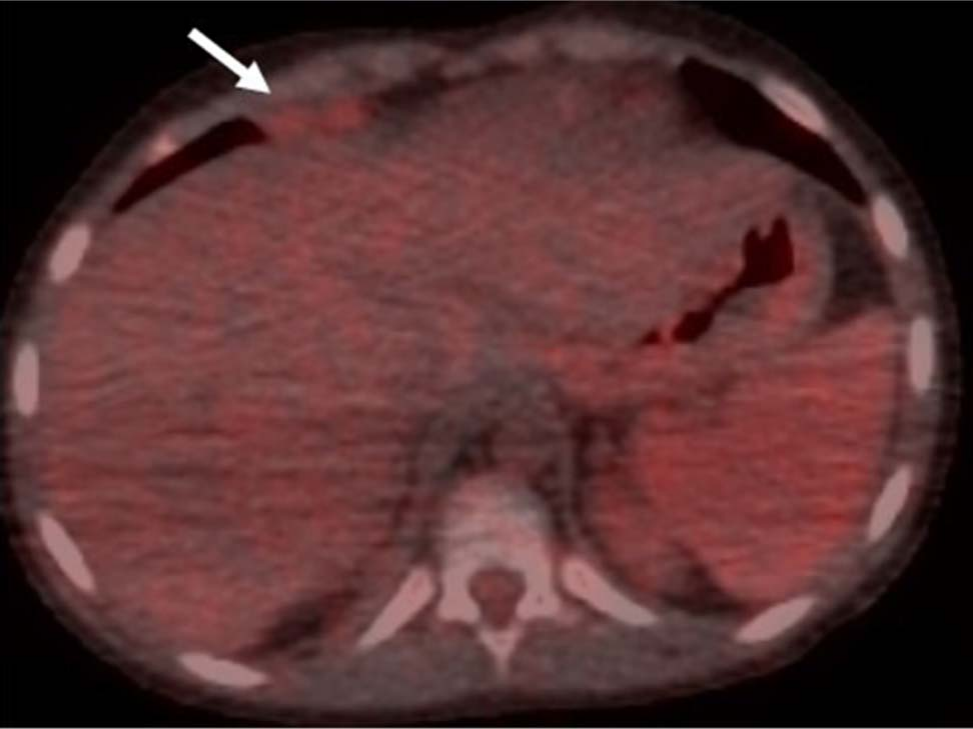
FDG PET/CT scan demonstrated increased metabolic activity of the right pleural (SUV 2.3) lesions seen on contrast CT images. CT, computed tomography; PET, positron emission tomography; SUV, standardized uptake value.

**Figure 4.**
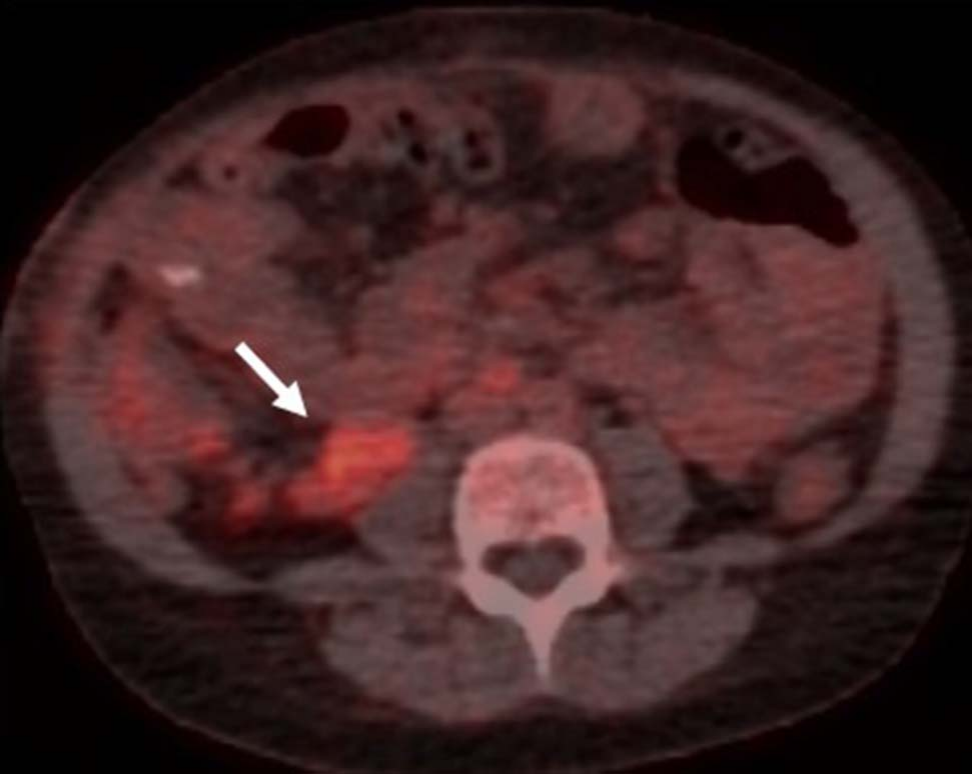
FDG PET/CT scan demonstrated increased metabolic activity of the retroperitoneal (SUV 4.7) lesions seen on contrast CT images. CT, computed tomography; PET, positron emission tomography; SUV, standardized uptake value.

**Figure 5.**
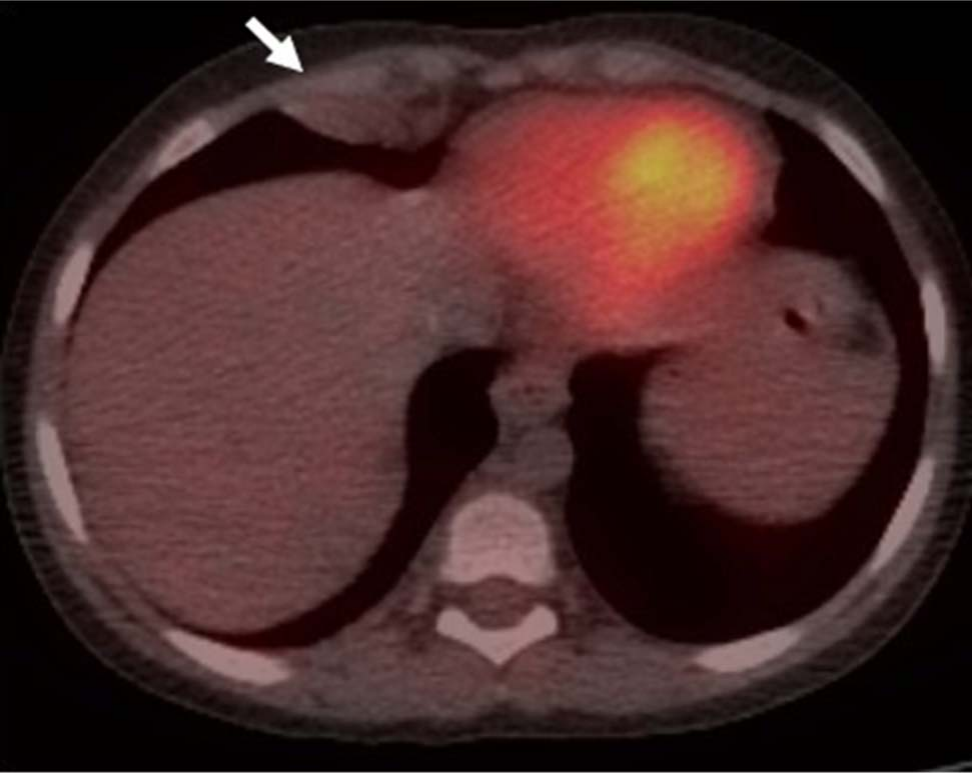
FDG PET/CT scan demonstrated interval-resolved FDG activity of the right pleural lesions 6 weeks after dose reduction of immunosuppression therapy. CT, computed tomography; PET, positron emission tomography.

**Figure 6.**
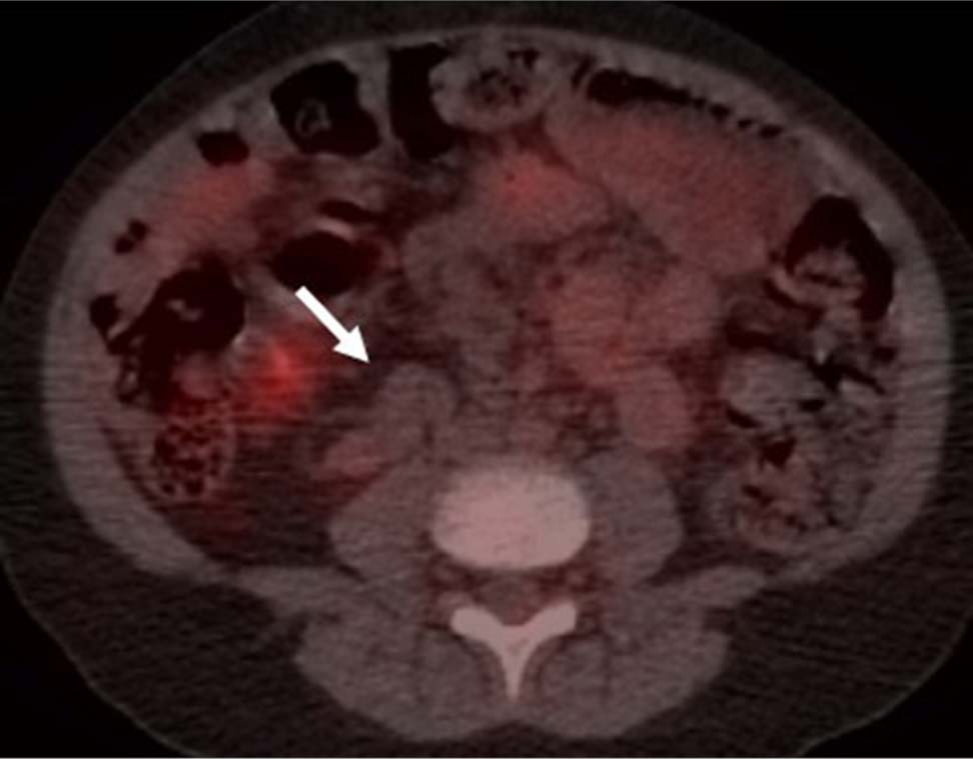
FDG PET/CT scan demonstrated interval-resolved FDG activity of the retroperitoneal lesions 6 weeks after dose reduction of immunosuppression therapy. CT, computed tomography; PET, positron emission tomography; SUV, standardized uptake value.

Within 24 hours, her respiratory status rapidly deteriorated. Repeat chest X-ray showed worsening right lower pleural effusion. Therapeutic thoracentesis was performed with improvement in symptoms. Pleural fluid was negative for infections (including EBV and CMV), and cytopathology was negative for malignancy. Chest computed tomography (CT) demonstrated 2 pleural-based masses—a 1.0 × 1.1 × 2.6 cm right supradiaphragmatic pleural lesion and a 1.7 × 3.3 × 1.5 cm right retroperitoneal lymph node concerning for PTLD (Figures 1 and 2). A fluorodeoxyglucose (FDG) positron emission tomography (PET) CT showed increased FDG uptake in the right supradiaphragmatic pleural lesion and enlarged retroperitoneal lymph nodes, suggestive of PTLD. Based on these findings, treatment of PTLD was initiated with reduction of her immunosuppression by reducing the tacrolimus dose from 2 mg twice daily to 0.5 mg twice daily, a reduction of 75% (Figures 3 and 4). Within 4 weeks, her neutrophil count normalized and she no longer suffered from intermittent fevers. A repeat FDG PET/CT scan 6 weeks later demonstrated decreased or resolved FDG uptake in multiple FDG-avid pleural and abdominal lymph nodes (Figures 5 and 6). Follow-up revealed stable neutrophil counts and no more symptoms. A follow-up PET/CT scan 3 months later showed no evidence of recurrent hypermetabolic activity.

## DISCUSSION

PTLD is a serious and life-threatening form of lymphoproliferative disease that occurs as a result of immunosuppression after organ transplantation. It is characterized by the uncontrolled proliferation of lymphocytes due to decreased cytotoxic T-lymphocyte and natural killer cell function.^1,3–5^ In pediatric liver transplant patients, PTLD occurs in approximately 5%–10% of cases, with a 2–4-fold higher risk compared with adult liver transplant recipients (3). The presence of EBV infection is found in 60%–80% of PTLD cases, highlighting the significant role of latent EBV infection in the development of the disease.^1,3,4,6^ In pediatric patients, even higher rates of EBV incidence have been reported.^3^ Notably, the combination of an EBV-seronegative recipient and EBV-seropositive donor increases the risk of PTLD development.^3,5^ In addition, the presence of CMV infection is associated with a higher risk of PTLD.^2,3,5^ Lung, heart, and multiorgan transplant recipients have a higher incidence of PTLD compared with liver and kidney transplant recipients, likely because of the increased intensity and duration of immunosuppressive therapy required in these cases.^5^ The age of the patient at the time of transplantation is also a risk factor, with patients younger than 10 years having a higher incidence of PTLD. This is because they are more likely to be EBV-seronegative at the time of transplantation, making them more susceptible to acute EBV and CMV infections early in the post-transplant period, combined with the immaturity of the recipient's immune system.^5,7,8^ The specific type of immunosuppression used also appears to be a contributing factor because certain agents such as antithymocyte globulin, calcineurin inhibitors, anti-CD3 (OKT3), tacrolimus, and cyclosporine have been associated with an increased risk of PTLD.^5^ Finally, genetic factors including variations in cytokine genes such as interleukin-10, interleukin-6, and interferon-gamma may influence the predisposition to PTLD.^5^

Time from transplantation to PTLD has a bimodal distribution in pediatrics, with one peak in the first year after transplantation (early PTLD) and another in the second to third year. Almost all early PTLDs are EBV-associated and frequently present atypically with extranodal or graft organ involvement.^3^ PTLD has been reported to involve the transplanted organ in heart, lung, and liver transplants in approximately half of cases.^5^ Late-onset PTLD is more likely to present with classical lymphoma.^3^ The symptoms are widely variable and can include classic B symptoms while extranodal involvement can involve the gastrointestinal tract, lungs, bone marrow, and central nervous system.^1–3^ Patients may present with nonspecific symptoms including malaise, anorexia, lymphadenopathy, tonsillar hypertrophy, abdominal masses, abdominal pain, upper airway obstruction, diarrhea, and hepatosplenomegaly. Laboratory data may reveal anemia, leukopenia, thrombocytopenia, hypoalbuminemia, abnormal liver enzymes, and protein-losing enteropathy.^2,3^

Histopathological examination of the tissue is considered gold standard for PTLD diagnosis, although the diagnosis is also based on a combination of histologic, immunophenotypic, radiologic, and genetic studies interpreted in the specific clinical context.^6,7^ Per definition, every lymphoid malignancy arising after transplantation is classified as PTLD.^3^ The World Health Organization Classification of Tumors of Hematopoietic and Lymphoid Tissue 2017 includes 6 types of morphological PTLDs: plasmacytic hyperplasia, infectious mononucleosis-like, florid follicular hyperplasia, polymorphic, monomorphic (B-cell or T/NK-cell types), and classical Hodgkin lymphoma PTLD.^1^ This classification applies in pediatric and adult patients.^8^ Workup in post-transplant recipients should be undertaken in the setting of a high index of suspicion such as a history of B symptoms, EBV viremia, and other risk factors for disease development mentioned above.^5^

Radiological evidence of a mass with PET scanning showing metabolically active areas may also support the diagnosis. Some studies have reported that FDG-PET/CT imaging is highly sensitive for the diagnosis of PTLD with an excellent ability to differentiate PTLD from other diagnoses.^9^ A study involving 34 patients examined the performance of FDG-PET/CT in identifying PTLD. The results indicated that FDG-PET/CT had an 85% sensitivity, 90% specificity, positive predictive value of 83%, and negative predictive value of 92%. The study demonstrated good interobserver agreement, suggesting that FDG-PET/CT imaging showed promising diagnostic capability in patients with PTLD. However, further research, particularly in pediatrics, is needed before considering its inclusion in PTLD diagnosis guidelines. Notably, the study highlighted false-positive results in FDG-PET/CT scans, which were attributed to infections and other malignancies.^10^

Owing to the clinicopathologic heterogeneity of the disease, there is no single unified treatment approach. The mainstay of treatment is reduction of immunosuppression. Other treatment options include rituximab monoclonal antibody given combined or in sequence with combination chemotherapy while surgery and/or radiation therapy may be considered in select cases.^5^ Assuming that active EBV replication contributes to the pathogenesis of EBV-positive PTLD, ganciclovir and acyclovir have been tried for the treatment of PTLD. Effectiveness has not been shown, however, because the latently EBV-infected tumor cells do not express the viral protein kinase that is essential for the drugs' activity.^11^ Prophylaxis with antivirals, however, demonstrated a risk reduction ranging between 38% and 44%, and the benefit was more prominent when EBV prophylaxis was given in the first year after transplant.^5^

Based on our literature review, this is the first reported case of PTLD involving the pleura reported in pediatrics. There is only one other case report wherein a 57-year-old patient with PTLD presented with pleural masses resulting in pleural effusions. Pleural fluid cytology was also unrevealing in his case, and the diagnosis was confirmed by pleural mass cytopathologic examination.^12^ Detection of primary PTLD by cytopathology in the pleural fluid in the absence of solid-organ involvement is also exceedingly rare, having been detected in 10 case reports in the literature.^13^ Interestingly, our patient's pleural fluid cytopathological examination was unrevealing despite the presence of metabolically active pleural lesions on her FDG PET/CT. In our patient's case, it was challenging to conduct histopathological examination of the metabolically active lesions because of the difficulty of accessing them and the associated high risks. Despite it not being the standard of care, a diagnosis of PTLD was made based on the patient's clinical presentation, risk factors of PTLD development, and imaging. These risk factors included a history of EBV viremia, young age, unexplained fevers, leukopenia, sudden-onset severe noninfectious pleural effusions, lymphadenopathy, pleural masses, and metabolically active lesions observed on PET/CT imaging. The resolution of symptoms and the decrease in metabolic activity in the lymph nodes and pleural lesions after a reduction in immunosuppression further supported the PTLD diagnosis. It is important to note the potential for false-positive results with FDG PET/CT imaging, such as an unidentified infectious cause leading to FDG-avid lymphadenopathy or other types of malignancies.^10^ However, in the case of infectious/inflammatory processes, FDG uptake typically intensifies after the reduction of the immunosuppression regimen because of the restored immune capability, which is contrary to what is observed in our patient.^14^ As for other possible malignancies, the reduction of immunosuppression generally does not affect the tumor's metabolic activity, although it cannot be completely ruled out.

There is one other case report wherein a patient with PTLD presented with pleural-based masses resulting in pleural effusions. Examination of the pleural fluid did not demonstrate malignancy or EBV viremia in both cases. In patients with risk factors, maintaining a high index of suspicion of PTLD located in the pleura depending on clinical history and the presence of pleural effusion is important. PTLD may be underrepresented in pleural fluid cytology samples. FDG PET/CT scan is a sensitive modality that may aid in the diagnosis of the disease.

## DISCLOSURES

Author contributions: E. Nessim Kostandy followed the patient closely and reviewed her chart in detail to produce the manuscript and is the article guarantor. D. Wan provided the best radiology images and descriptions for the case based on his expertise in nuclear medicine. E. Imseis managed the patient and reviewed the manuscript for accuracy.

Financial disclosure: None to report.

Informed consent was obtained for this case report.
